# Metabolic engineering of *Saccharomyces cerevisiae* for the *de novo* production of psilocybin and related tryptamine derivatives

**DOI:** 10.1016/j.ymben.2019.12.007

**Published:** 2020-07

**Authors:** N. Milne, P. Thomsen, N. Mølgaard Knudsen, P. Rubaszka, M. Kristensen, I. Borodina

**Affiliations:** The Novo Nordisk Foundation Center for Biosustainability, Technical University of Denmark, 2800 Kgs, Lyngby, Denmark

**Keywords:** Psilocybin, Tryptamine derivatives, *Saccharomyces cerevisiae*, Metabolic engineering, *Psilocybe cubensis*, Baeocystin, Norbaeocystin, Aeruginascin

## Abstract

Psilocybin is a tryptamine-derived psychoactive alkaloid found mainly in the fungal genus *Psilocybe*, among others, and is the active ingredient in so-called “magic mushrooms”. Although its notoriety originates from its psychotropic properties and popular use as a recreational drug, clinical trials have recently recognized psilocybin as a promising candidate for the treatment of various psychological and neurological afflictions. In this work, we demonstrate the *de novo* biosynthetic production of psilocybin and related tryptamine derivatives in *Saccharomyces cerevisiae* by expression of a heterologous biosynthesis pathway sourced from *Psilocybe cubensis*. Additionally, we achieve improved product titers by supplementing the pathway with a novel cytochrome P450 reductase from *P. cubensis.* Further rational engineering resulted in a final production strain producing 627 ± 140 mg/L of psilocybin and 580 ± 276 mg/L of the dephosphorylated degradation product psilocin in triplicate controlled fed-batch fermentations in minimal synthetic media. Pathway intermediates baeocystin, nor norbaeocystin as well the dephosphorylated baeocystin degradation product norpsilocin were also detected in strains engineered for psilocybin production. We also demonstrate the biosynthetic production of natural tryptamine derivative aeruginascin as well as the production of a new-to-nature tryptamine derivative *N-*acetyl-4-hydroxytryptamine. These results lay the foundation for the biotechnological production of psilocybin in a controlled environment for pharmaceutical applications, and provide a starting point for the biosynthetic production of other tryptamine derivatives of therapeutic relevance.

## Highlights

• *De novo* production of psilocybin in *S. cerevisiae*.
• Expression of a novel cytochrome P450 reductase from *P. cubensis* significantly boosts production.
• Rational metabolic engineering results in 627 mg/L psilocybin production.
• Production of natural and new-to-nature tryptamine derivatives demonstrated including norbaeocystin, baeocystin, and aeruginascin.

## 1 Introduction

**Fig. 1:**
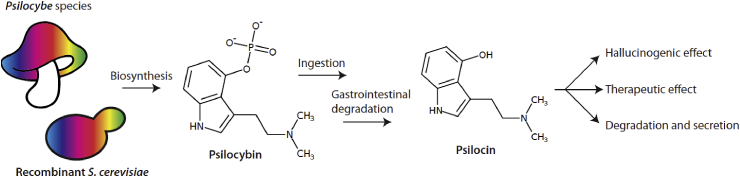
**Psilocybin route of administration in humans**. After biosynthesis in *Psilocybe* mushroom species (or in this case *S. cerevisiae*), psilocybin is typically administered by oral ingestion. Upon consumption, psilocybin acts as a prodrug where alkaline phosphatases, non-specific esterases, or the acidic conditions in the stomach convert the molecule to the bioactive psilocin. Psilocin then exerts its psychotropic or therapeutic effect by crossing the blood-brain barrier and interacting with serotonin receptors. Psilocin is eventually removed from the body via glucuronidation and excretion through the kidneys (Manevski et al., 2010).

Psilocybin is an alkaloid produced mainly by mushrooms from the basidiomycete genus Psilocybe, popularly known as “magic mushrooms”. Psilocybin itself is not psychoactive - rather it is the dephosphorylated derivative psilocin that causes the hallucinogenic effect. Psilocybin is rapidly dephosphorylated to psilocin following ingestion in the mucosa by alkaline phosphatases and nonspecific esterases (Tylš et al., 2014) (Fig. 1). Psilocin is structurally similar to human neurotransmitters such as serotonin, and it is therefore not surprising that psilocin has been shown to bind to over 15 human serotonin-related receptors (Ray, 2010).

In 1957, Robert G. Wasson published a popular science article in LIFE magazine describing his experiences with “divine mushrooms” from a remote, indigenous Mexican village (Wasson, 1957). A year later, the psychoactive compound in the mushrooms was found to be psilocybin. This discovery sparked an academic interest in the use of psilocybin as a drug for pharmacological and therapeutic clinical research, lasting through the 1960's. Psilocybin was however classified as a Schedule I drug in the US in 1970 due to its popularity as a recreational drug and its perceived harm, putting a damper on research activities until the late 1990's, when the drug became a popular research topic once again (Tylš et al., 2014). Recently, thanks in part to changing views on the criminalization of illicit drug use, the therapeutic potential of several recreational drugs has been investigated. Psilocybin in particular has seen success in clinical trials for the treatment of different psychological and neurological afflictions. At the time of writing, 40 clinical trials had been completed or were ongoing for psilocybin according to “clinicaltrials.gov”. Preliminary results suggest that psilocybin-assisted treatment may be a good candidate for managing substance addiction (Bogenschutz et al., 2015; Riaz et al., 2016), anxiety in terminally ill patients (Grob et al., 2011), cluster headaches (Tylš et al., 2014), and treatment-resistant depression (Carhart-Harris et al., 2018). According to these trials and a number of toxicological studies, psilocybin has a very good safety profile (Tylš et al., 2014), showing no sign of toxicity in isolated organs of rats and pigs, and a LD_50_ of 280–285 mg/kg in rats and mice (Johnson et al., 2008). Psilocybin seems to be a particularly interesting candidate for “treatment resistant depression” - a term applied to the 13% of patients with Major Depressive Disorder (MDD) who relapse, in spite of four rounds of traditional treatment (Rush et al., 2006). Approximately 16 million Americans carried the MDD diagnosis in 2016, indicating a large number of people with untreated mental illness (Tice, 2017). Recently, the Food and Drug Administration (FDA) granted the company COMPASS Pathways “Breakthrough therapy” status for the use of psilocybin in treatment-resistant depression, hastening regulatory approval and decreasing time to market (Haridy, 2018).

With the impending approval of psilocybin as a pharmaceutical drug, the manufacture and supply should be considered. Unfortunately, the content of psilocybin and psilocin in psychedelic mushrooms is too low (0.2%–1% dry weight) to make extraction a commercially viable option (Tylš et al., 2014), with cultivation of mushrooms for extraction of its psychedelic compounds limited to recreational users only. Furthermore, the large variation in psilocybin content observed between different mushroom batches will present difficulties in generating a consistent product with a stable supply chain needed for large scale therapeutic application (Bigwood and Beug, 1982).

Psilocybin preparations for pharmaceutical and research use currently rely on chemical synthesis via a difficult and expensive process (Nichols and Frescas, 1999). Although the chemical synthesis of psilocybin has been improved since its initial discovery by Hoffman et al. in 1959, who achieved final yields of 20% semi-pure psilocybin, it continues to challenge chemists due both to the difficulty of the last synthetic step; the phosphorylation of psilocin (Nichols and Frescas, 1999) as well as the stereospecific 4-hydroxylation of the aromatic ring (Ullrich and Hofrichter, 2007). Recently the company COMPASS pathways developed and patented a new method for the chemical synthesis of psilocybin, and while it improves on previous methods with an overall yield of 75%, it uses expensive 4-hydroxyindole as a starting substrate (Londesbrough et al., 2019) resulting in high production costs which may limit its application.

Biotechnological production of psilocybin is an attractive alternative as it offers the high yields and purity required for pharmaceutical applications, while allowing for a simple and cost-effective production process from simple starting substrates such as glucose. Furthermore, unlike chemical synthesis, microbial cell-factories can be rapidly adapted to produce a variety of valuable tryptamine derivatives, thereby expanding the utility of this approach. To that end, the biosynthetic pathway of psilocybin from the psychedelic mushroom *P. cubensis* was recently elucidated (Fricke et al., 2017), and heterologous production in the filamentous fungi *Aspergillus nidulans* was established with titers reaching 110 mg/L (Hoefgen et al., 2018). Production of psilocybin has also recently been demonstrated in *Escherichia coli* (*E. coli*) by the *in vivo* bioconversion of added substrates 4-hydroxyindole, serine and methionine, with a titer of 1.16 g/L reported (Adams et al., 2019). While the titer demonstrates the feasibility of biotransformation, the addition of multiple expensive starting substrates will likely make it difficult to compete on price with chemical synthesis from the same expensive precursor.

**Fig. 2:**
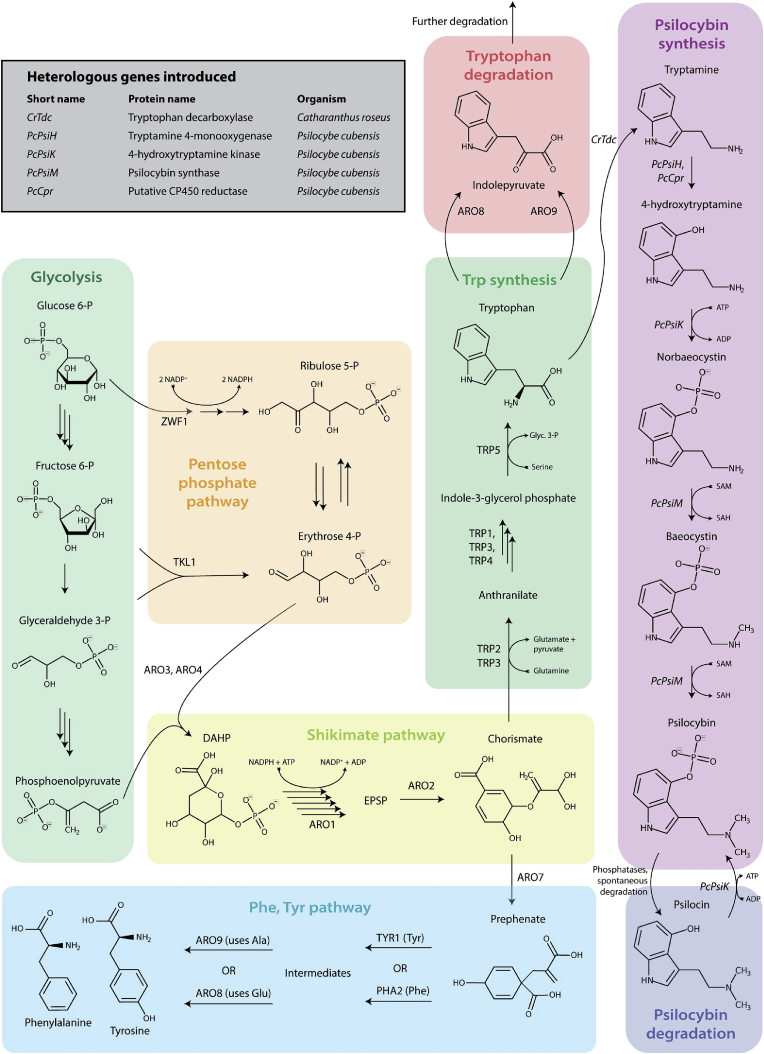
**Psilocybin biosynthesis in *S. cerevisiae***. The heterologous biosynthetic pathway begins with the native production of tryptophan, which itself is derived from metabolites produced via glycolysis, the pentose phosphate pathway, and the shikimate pathway. DAHP, 3-deoxy-D-arabinoheptulosonate 7-phosphate; EPSP, 5-enolpyruvoyl-shikimate 3-phosphate; Glyc. 3-P, glyceraldehyde 3-phosphate; Ala, alanine; Glu, glutamate; Tyr, tyrosine; Phe, phenylalanine. Multiple arrows represent multiple enzymatic reactions grouped for simplicity.

To achieve cost-competitive large-scale fermentative production of psilocybin according to GMP regulations we postulated that *S. cerevisiae* might make a more attractive production host due to its long use in industrial production as well as the fact that it naturally produces very few secondary metabolites or other tryptophan derivatives, thereby facilitating simple downstream processing and purification (Borodina and Nielsen, 2014). Furthermore, unlike prokaryotes like *E. coli*, *S. cerevisiae* can functionally express cytochrome P450 enzymes required for the conversion of tryptamine to 4-hydroxytryptamine (Fig. 2), thereby enabling *de novo* production from glucose via the native metabolite tryptophan, and circumventing the need to add the expensive substrate 4-hydroxyindole (approx. $288 USD/g from Sigma Aldrich) as previously reported (Adams et al., 2019), resulting in a significant reduction in production costs. Here we report the *de novo* biosynthetic production of psilocybin in *S. cerevisiae* through rational metabolic engineering as outlined in Fig. 2.

## 2 Materials and methods

### 2.1 Strains media and maintenance

All *S. cerevisiae* strains used in this study (Table 1) were derived from the CEN.PK strain family background (Entian and Kötter, 2007; Nijkamp et al., 2012). Frozen stocks of *E. coli* and *S. cerevisiae* were prepared by addition of glycerol (30% (v/v)) to exponentially growing cells and aseptically storing 1 mL aliquots at −80ᵒC. Cultures were grown in synthetic medium according to the following recipes. Synthetic medium was prepared with 7.5 g/L (NH_4_)_2_SO_4_, 14.4 g/L KH_2_PO_4_, 0.5 g/L MgSO_4_7H_2_O and appropriate growth factors (Pronk, 2002). Synthetic drop-out media minus uracil supplementation (SC-Ura) was prepared with 3 g/L synthetic complete minus uracil powder (Sigma-Aldrich) and 5 g/L (NH_4_)_2_SO_4_. YP medium was prepared with 10 g/L yeast extract and 20 g/L peptone. In all cases unless stated otherwise, 20 g/L glucose was added. Media was supplemented with 200 mg/L G418 and 100 mg/L nourseothricin when required. *E. coli* strains were grown in Luria–Bertani (LB) media and supplemented with 100 mg/L ampicillin when required. Agar plates were prepared as described above but with the addition of 20 g/L agar.

**Table 1:** Strains used in this study. Unless stated otherwise, relevant genes are expressed from strong constitutive promoters.

Name	Parental strain	Added DNA element	Relevant genotype	Source
CEN.PK113-5D	–	–	MATa *Δura3 HIS3 LEU2 TRP1 MAL2-8c SUC2*	Entian and Kötter (2007)
ST8251	CEN.PK113-5D	pCfB2312	MATa *Δura3 HIS3 LEU2 TRP1 MAL2-8c SUC2 + pCfB2312 (Cas9)*	Jessop-Fabre et al. (2016)
ST8940	ST8251	pCfB8794	MATa *Δura3 HIS3 LEU2 TRP1 MAL2-8c SUC2 XII-5*:: *PcPsiM-PcPsiK*	This study
ST8983	ST8940	BB3923	MATa *Δura3 HIS3 LEU2 TRP1 MAL2-8c SUC2 XII-5*:: *PcPsiM-PcPsiK XI-3*:: *CrTdc-PcPsiH*	This study
ST9016	ST8983	BB3939	MATa *Δura3 HIS3 LEU2 TRP1 MAL2-8c SUC2 XII-5*:: *PcPsiM-PcPsiK XI-3*:: *CrTdc-PcPsiH XI-1*:: *PcCpr*	This study
ST9020	ST8983	BB3940	MATa *Δura3 HIS3 LEU2 TRP1 MAL2-8c SUC2 XII-5*:: *PcPsiM-PcPsiK XI-3:: CrTdc-PcPsiH XI-1:: pTEF2→PcCpr*	This study
ST9109	ST8983	BB3953	MATa *Δura3 HIS3 LEU2 TRP1 MAL2-8c SUC2 XII-5:: PcPsiM-PcPsiK XI-3:: CrTdc-PcPsiH XI-2:: CYB5-AtAtr2*	This study
ST9179	ST9016	PR-23852	MATa *Δura3 HIS3 LEU2 TRP1 MAL2-8c SUC2 XII-5:: PcPsiM-PcPsiK XI-3:: CrTdc-PcPsiH XI-1:: PcCpr ric1::*	This study
ST9316	ST9179	BB4020	MATa *Δura3 HIS3 LEU2 TRP1 MAL2-8c SUC2 XII-5:: PcPsiM-PcPsiK XI-3:: CrTdc-PcPsiH XI-1:: PcCpr ric1:: X-4:: ARO1-ARO2*	This study
ST9318	ST9316	pCfB9074	MATa *Δura3 HIS3 LEU2 TRP1 MAL2-8c SUC2 XII-5:: PcPsiM-PcPsiK XI-3:: CrTdc-PcPsiH XI-1:: PcCpr ric1:: X-4:: ARO1-ARO2 XII-4:: ARO4*^*K229L*^*-TRP2*^*S65R,S76L*^	This study
ST9326	CEN.PK113-5D	pCfB255	MATa *Δura3 HIS3 LEU2 TRP1 MAL2-8c SUC2 X-2:: URA3*	This study
ST9327	ST8983	pCfB255	MATa *Δura3 HIS3 LEU2 TRP1 MAL2-8c SUC2 XII-5:: PcPsiM-PcPsiK XI-3:: CrTdc-PcPsiH X-2:: URA3*	This study
ST9328	ST9016	pCfB255	MATa *Δura3 HIS3 LEU2 TRP1 MAL2-8c SUC2 XII-5:: PcPsiM-PcPsiK XI-3:: CrTdc-PcPsiH XI-1:: PcCpr X-2:: URA3*	This study
ST9329	ST9020	pCfB255	MATa *Δura3 HIS3 LEU2 TRP1 MAL2-8c SUC2 XII-5:: PcPsiM-PcPsiK XI-3:: CrTdc-PcPsiH XI-1:: pTEF2→PcCpr X-2:: URA3*	This study
ST9330	ST9109	pCfB255	MATa *Δura3 HIS3 LEU2 TRP1 MAL2-8c SUC2 XII-5:: PcPsiM-PcPsiK XI-3:: CrTdc-PcPsiH XI-2:: CYB5-AtAtr2 X-2:: URA3*	This study
ST9334	ST9016	pCfB9013	MATa *Δura3 HIS3 LEU2 TRP1 MAL2-8c SUC2 XII-5:: PcPsiM-PcPsiK XI-3:: CrTdc-PcPsiH XI-1:: PcCpr Ty-4::PcPsiK*	This study
ST9335	ST9016	pCfB8796	MATa *Δura3 HIS3 LEU2 TRP1 MAL2-8c SUC2 XII-5:: PcPsiM-PcPsiK XI-3*:: *CrTdc-PcPsiH XI-1:: PcCpr Ty-4::PcPsiM*	This study
ST9337	ST8251	pCfB8881	MATa *Δura3 HIS3 LEU2 TRP1 MAL2-8c SUC2 XI-3:: CrTdc*	This study
ST9346	ST9337	pCfB9073	MATa *Δura3 HIS3 LEU2 TRP1 MAL2-8c SUC2 XI-3:: CrTdc XI-1:: PcCpr-PcPsiH*	This study
ST9441	ST9346	pCfB8796	MATa *Δura3 HIS3 LEU2 TRP1 MAL2-8c SUC2 XI-3:: CrTdc XI-1:: PcCpr-PcPsiH Ty-4:: PcPsiM*	This study
ST9442	ST9346	pCfB9111	MATa *Δura3 HIS3 LEU2 TRP1 MAL2-8c SUC2 XI-3:: CrTdc XI-1:: PcCpr-PcPsiH Ty-4:: BtAANAT*	This study
ST9482	ST9318	pCfB8796	MATa *Δura3 HIS3 LEU2 TRP1 MAL2-8c SUC2 XII-5*:: *PcPsiM-PcPsiK XI-3*:: *CrTdc-PcPsiH XI-1*:: *PcCpr ric1*:: *X-4*:: *ARO1-ARO2 XII-4*:: *ARO4*^*K229L*^*-TRP2*^*S65R,S76L*^*Ty-4*:: *PcPsiM*	This study
ST9649	ST9327	BB4335	MATa *Δura3 HIS3 LEU2 TRP1 MAL2-8c SUC2 XII-5*:: *PcPsiM-PcPsiK XI-3*:: *CrTdc-PcPsiH XI-1*:: *NCP1 X-2*:: *URA3*	This study

### 2.2 Plasmid and strain construction

*E. coli* DH5α was used for all plasmid cloning and propagation. Single integration plasmids were constructed using the EasyClone-MarkerFree system (Jessop-Fabre et al., 2016), and multiple integration plasmids were constructed using a modified version of the EasyCloneMulti system (Maury et al., 2016) using a backbone plasmid where multiple integration into the *S. cerevisiae* genome was achieved using a *Kluyveromyces lactis* URA3 gene (KlURA3) under control of a truncated 10bp KlURA3 promoter. Heterologous genes were codon-optimized for expression in *S. cerevisiae* using the JCat algorithm (Grote et al., 2005) and ordered as synthetic gene strings (GeneArt). DNA was transformed into *S. cerevisiae* using the LiAc method according to (Gietz and Woods, 2002). Successful genomic integration of overexpression cassettes was confirmed by diagnostic PCR, successful integration of multi-copy Ty integrative cassettes was confirmed by screening at least six independent clones for increased production of a metabolite of interest. A list of all primers, biobricks and plasmids used in this study can be found in the supplementary data (Supplementary Tables 1, 2 and 3).

### 2.3 Cultivation and analysis

*E. coli* cells were cultured at 37 °C with shaking at 300 rpm. *S. cerevisiae* cells were cultured at 30 °C with shaking at 300 rpm. For micro-titer plate (MTP) cultivation of psilocybin producing *S. cerevisiae* strains, cells were inoculated from a 400 μL synthetic media pre-culture into 500 μL synthetic media to a starting OD600 of 0.1 in a 96-deep well microtiter plate with air-penetrable lid (EnzyScreen, NL) and incubated for 72 h with shaking at 300 rpm. When required, uracil was added to a final concentration of 200 mg/L.

Controlled fed-batch fermentation was carried out in 1 L bioreactors (BIOSTAT® Q plus, Sartorius, Goettingen, Germany) equipped with measurement probes for pH, dissolved oxygen (DO) and temperature. During fermentation, off-gas CO_2_, O_2_ and ethanol were monitored continuously (Prima BT MS; Thermo Scientific), and data acquisition was achieved using the Lucullus software (Securecell AG, Switzerland). Seed culture was prepared by cultivating the strain for 48 h at 30 ᵒc in 50 mL of synthetic media containing; 3 g/L KH_2_PO_4_, 0.5 g/L MgSO_4_.7H_2_O, 5 g/L (NH_4_)_2_SO_4_, 20 g/L glucose and appropriate vitamins and trace elements (Pronk, 2002). The seed culture was washed and resuspended in fresh synthetic media and inoculated into bioreactors containing 300 mL of synthetic media as described above but with addition of 0.3 mL/L Antifoam 204 (Sigma Aldrich) to a starting OD_600_ of 0.1. Fermentation was carried out at 30 °C, with 1 vvm aeration and the pH controlled at 5.0 by addition of a 12% NH_4_OH solution. The O_2_ and CO_2_ concentrations in the off-gas were monitored, and upon observing a sharp increase in O_2_ and corresponding decrease in CO_2_ (indicating complete consumption of glucose), fed-batch phase was initiated at a starting feed rate of 0.45 g/h with an exponential increase of 0.02 h^−1^), fed-batch phase was initiated at a starting feed rate of 0.45 g/h with an exponential increase of 0.02 h^−1^. Feed media contained; 30 g/L KH_2_PO_4_, 6.3 g/L MgSO_4_.7H_2_O, 50 g/L (NH_4_)_2_SO_4_, 0.35 g/L Na_2_SO_4_, 500 g/L glucose, 1 mL/L Antifoam 204 and a 10 fold higher vitamin and trace element concentration (Pronk, 2002). During cultivation, the DO level was kept above 40% by cascaded control of the stirrer speed and air input flow rate.

Extraction of extracellular tryptamine derived metabolites for analysis was performed as follows. Cell culture broth was supplemented with 100% acetonitrile at a ratio of 1:1, vortexed thoroughly then centrifuged at 3000 g for 5 min. The resulting supernatant was further diluted in 50% acetonitrile (v/v) if required and analyzed using LC-MS with the following conditions; High resolution LC-MS measurements were carried out on a Dionex UltiMate 3000 UHPLC (Thermo Fisher Scientific, US), connected to an Orbitrap Fusion Mass Spectrometer (Thermo Fisher Scientific, US). The UHPLC was equipped with a SeQuant zic-Hilic column (Merck KGaA), 15 cm x 2.1 mm, 3 μm. The temperature was 35 °C and the flow rate 0.5 mL/min. The system was running an isocratic gradient with a mobile phase consisting of 20% 10 mM ammonium formate (pH 3) and 80% acetonitrile, with 0.1% formic acid. The samples were passed on to the MS equipped with a heated electrospray ionization source (HESI) in positive-ion mode with sheath gas set to 50 (a.u.), aux gas to 10 (a.u.) and sweep gas to 1 (a.u.). The cone and probe temperature were 325 °C and 350 °C, respectively, and spray voltage was 3500 V. Scan range was 100–800 Da and time between scans was 50 ms. Psilocybin, psilocin and tryptamine authentic analytical standards were obtained from Sigma-Aldrich and used to quantify production in engineered strains.

Extraction of all other extracellular metabolites was performed by centrifuging cell cultures and collecting the resulting supernatant which were analyzed for quantification of ethanol (and other metabolites) as follows. Metabolites were detected and quantified using a high performance liquid chromatography (HPLC) Agilent 1100 series with a refractive index detector and a Bio-Rad Aminex HPX-87H column (300 mm × 7.8 mm) with 5 mM H2SO4 as an eluent at a flow rate of 0.6 mL/min with column oven temperature set to 50 °C. To protect the HPX-87H column from contamination and foreign particles, a guard column was fitted to the system.

## 3 Results

### 3.1 Expression of a heterologous psilocybin biosynthetic pathway in *S. cerevisiae*

Heterologous genes encoding the catalytic enzymes for psilocybin biosynthesis were introduced into *S. cerevisiae* strain ST8251 (CEN.PK113-5D + Cas9) (Fig. 2). The biosynthetic production of psilocybin starts with L-tryptophan, which is converted into tryptamine by tryptophan decarboxylase. *Catharanthus roseus* (*C. roseus*) tryptophan decarboxylase (*CrTdc*) was used instead of the *P. cubensis* variant (*PsiD*), due to its previously confirmed efficacy in *S. cerevisiae* (Brown et al., 2015). Tryptamine is next converted into 4-hydroxytryptamine by a cytochrome P450 containing monooxygenase (*PcPsiH*). Cytochrome P450 enzymes are characterized by their dependency on a cytochrome P450 reductase (CPR), which facilitates electron transfer between NADPH and cytochrome P450 enzymes (Renault et al., 2014). In *S. cerevisiae*, this is encoded by NCP1. 4-hydroxytryptamine is next converted into norbaeocystin by a 4-hydroxytryptamine kinase encoded by *PcPsiK*, which facilitates the 4-O-phosphorylation reaction (Fricke et al., 2017). Finally, an *N*-methyltransferase encoded by *PcPsiM* mediates the iterative methyl transfer of norbaeocystin to baeocystin then to psilocybin. Due to relatively weak O-phosphorylation binding, psilocybin can be enzymatically (via endogenous phosphorylases) or spontaneously degraded to psilocin, however the 4-hydroxytryptamine kinase (*PcPsiK*) has been shown to also act on psilocin, resulting in a psilocybin-psilocin equilibrium reaction (Fricke et al., 2017).

**Fig. 3:**
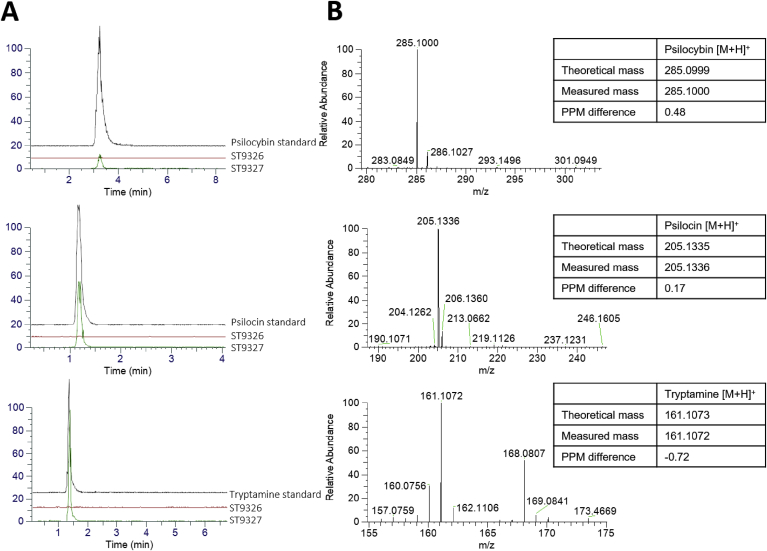
***De novo* psilocybin production in *S. cerevisiae*** (A). LC-MS chromatograms confirming psilocybin, psilocin and tryptamine production in ST9327 (psilocybin biosynthetic pathway) compared to wild-type control strain ST9326 using authentic analytical standards. (B). Corresponding mass spectra for psilocybin, psilocin and tryptamine peaks in ST9327.

The basic heterologous pathway was introduced into *S. cerevisiae* (resulting in strain ST9327), then, using an Orbitrap Fusion Mass Spectrometer and authentic analytical standards, successful production of psilocybin, as well as the pathway intermediate tryptamine, and the spontaneous degradation product psilocin was confirmed in micro-titer plate cultivation (Fig. 3). Quantification using calibration curves determined that ST9327 produced tryptamine, psilocybin and psilocin with titers of 120.3 ± 11.1 mg/L, 2.2 ± 0.7 mg/L, and 5.5 ± 0.7 mg/L, respectively, in duplicate micro-titer plate cultivation in synthetic media.

### 3.2 Expression of a novel cytochrome P450 reductase (CPR) from *P. cubensis* increases psilocybin production

**Fig. 4:**
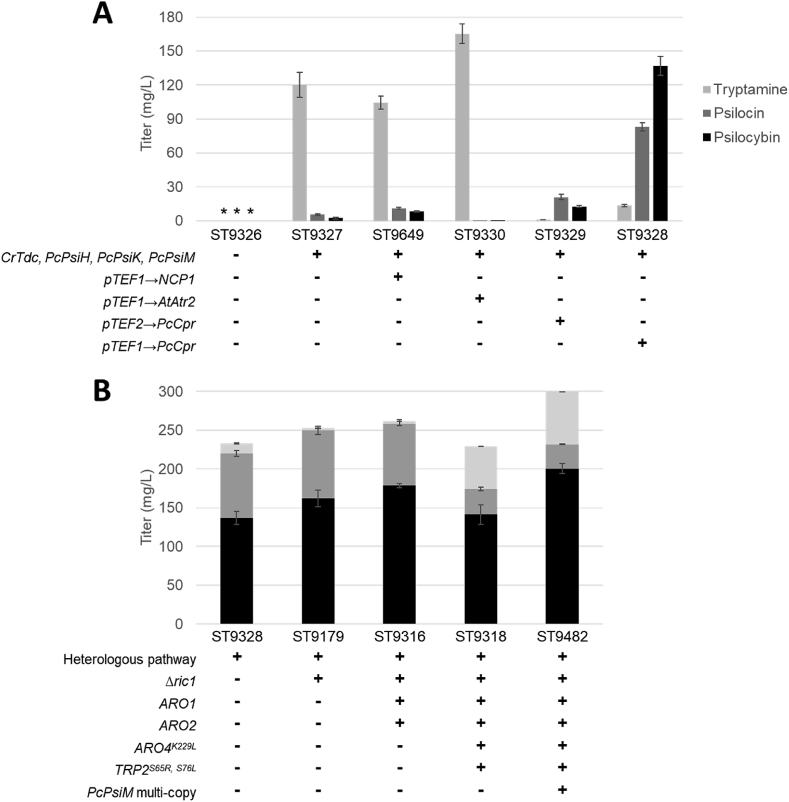
**Improved *De novo* psilocybin biosynthesis in *S. cerevisiae.*** (A). Introduction of the heterologous biosynthesis pathway and corresponding final titers in micro-titer plate cultivation. ST9326, wild-type parental strain; ST9327, psilocybin biosynthetic pathway (*CrTdc, PcPsiH, PcPsiK, PcPsiM*); ST9649, psilocybin biosynthetic pathway + NCP1 expressed from TEF1 promoter; ST9330, psilocybin biosynthetic pathway + *A. thaliana* CPR (*AtAtr2*) expressed from TEF1 promoter; ST9329, psilocybin biosynthetic pathway + *P. cubensis* CPR expressed from TEF2 promoter (*pTEF2→PcCpr*); ST9328, psilocybin biosynthetic pathway + *P. cubensis* CPR expressed from TEF1 promoter (*pTEF1→PcCpr*). (B). Iterative strain improvement to increase tryptophan availability and overcome rate-limiting reactions with resulting final titers in micro-titer plate cultivation. Gene names represent genes that were expressed from strong constitutive promoters. Strains were cultivated in synthetic media with 20 g/L glucose for 5 days and subjected to acetonitrile extraction and analysis by LC-MS. Media was supplemented with uracil when required. Data is presented as averages and standard deviations from biological duplicates. *; Not detected. Heterologous pathway; Strain expressing *Crtdc, PcpsiH, PcpsiK, PcpsiM* and *Pccpr* from the TEF1 promoter.

While psilocybin was successfully produced in yeast, the initial titers were low. Furthermore, analysis revealed the extracellular accumulation of tryptamine (120.3 ± 11.1 mg/L) indicating a significant limitation in the conversion of tryptamine to 4-hydroxytryptamine encoded by the cytochrome P450 gene *PcpsiH*. Cytochrome P450 enzymes (CYP) belong to a superfamily of heme-containing monooxygenases and require a cytochrome P450 reductase (CPR) partner to deliver one or more electrons to reduce the heme-bound iron and oxidized substrates (Renault et al., 2014). While the detection of psilocybin indicated that the native *S. cerevisiae* CPR (Ncp1) could carry out this reduction, the accumulation of tryptamine suggested a sub-optimal interaction between the two enzymes. Due to the importance of the CYP-CPR interaction for optimal catalytic activity (Paddon et al., 2013; Renault et al., 2014), we attempted to find a more suitable reduction partner for *PcPsiH*. Accordingly, we identified a putative *P. cubensis* CPR (*PcCpr*) with a tBLASTx search of the *P. cubensis* genome (Fricke et al., 2017) using Ncp1 as a query sequence, which returned a single protein coding sequence with 42.2% homology to the Ncp1 amino acid sequence. To test the function of this putative CPR, we expressed it from the strong constitutive promoter pTEF1 and the medium-strength constitutive promoter pTEF2. An additional CPR from *Arabidopsis thaliana* (*A. thaliana*) *AtAtr2* was also tested with additional overexpression of the *S. cerevisiae* cytochrome *b*5 (CYB5) as this has previously been shown to enable functional expression of other CYP's (Li et al., 2016). Finally, because native *S. cerevisiae* Ncp1 had some capacity to act as a reductase partner for *PcPsiH*, the effect of overexpression of this enzyme from the pTEF1 promoter was also tested. While introduction of *Atatr2* into a strain containing the psilocybin biosynthetic pathway led to a decrease in titer (ST9330), and expression of *Pccpr* from the medium-strength TEF2 promoter only a small increase in titer (ST9329), expression of *Pccpr* from the strong TEF1 promoter produced a significant increase in psilocybin and psilocin titer (ST9328), reaching 137.1 ± 8.3 mg/L and 82.8 ± 3.7 mg/L respectively in micro-titer plate, representing a 29-fold increase over the parental strain (Fig. 4A). This not only confirmed the functional expression of a novel CPR from *P. cubensis*, but also highlighted the importance of investigating the compatibility and expression of a CYP with a CPR partner which was further demonstrated by the observation that overexpression of native CPR (NCP1) led to only a minor increase in titer.

### 3.3 Rational metabolic engineering of tryptophan precursor supply results in improved psilocybin titers

Functional implementation of *PcCpr* resulted in a significant increase in psilocybin and psilocin titers, and furthermore, significantly reduced extracellular accumulation of the first product in the heterologous pathway, tryptamine (13.3 ± 1.0 mg/L, Fig. 4A). Overall, this suggested that the heterologous pathway had sufficient capacity to convert available tryptophan to psilocybin and that we should next focus our efforts on boosting the native precursor supply. The shikimate pathway is an intensely studied pathway due to the diverse range of valuable products that can be derived from it. As such, there are a multitude of reports describing modifications that boost shikimate derived products (Averesch and Krömer, 2018). To boost psilocybin precursor supply, we introduced a series of modifications including overexpression of genes in the shikimate pathway (*ARO1*, *ARO2*), overexpression of feedback insensitive mutant genes encoding proteins in the shikimate pathway (*Aro4*^*K229L*^*, Trp2*^*S65R, S76L*^) (Graf et al., 1993; Luttik et al., 2008), and deletion of genes involved in regulation of the shikimate pathway (*RIC1*) (Suástegui et al., 2017). Iterative deletion of *RIC1* (resulting in strain ST9179) and subsequent overexpression of *ARO1* and *ARO2* (resulting in strain ST9316) lead to a significant increase in psilocybin and psilocin titers. While further overexpression of feedback insensitive *Aro4*^*K229L*^*, Trp2*^*S65R, S76L*^ (resulting in strain ST9318) resulted in a decrease in titer. In all three strains a significant increase in the LC-MS peak areas corresponding to norbaeocystin and baeocystin was observed compared to the parental strain containing the psilocybin biosynthetic pathway and optimized *Pccpr* expression (ST9328) (Supplementary Table 4). While in the absence of an authentic analytical standard it was not possible to completely identify and quantify the increased accumulation of these intermediates, it did strongly suggest the sub-optimal iterative N-methylation of norbaeocystin to baeocystin then to psilocybin, catalyzed by *PcPsiM*. In an attempt to improve enzyme activity, multiple copies of *PcpsiM* were introduced into the genome of ST9318 using a Ty-integrative vector based on (Maury et al., 2016) using a truncated 10bp *URA3* promoter to drive weakened expression of the *URA3* marker resulting in strain ST9482. Subsequent analysis of six independent clones gave consistent improvements in titer, indicating that clonal variation from semi-random genomic integration was minimal. Introducing multiple copies of *PcpsiM* resulted in a significant increase in production with final psilocybin and psilocin titers reaching 200.5 ± 6.5 mg/L and 31.4 ± 0.1 mg/L respectively (Fig. 4B).

Due to the high concomitant production of psilocin observed in all strains tested, we attempted to reduce this accumulation by introducing multiple copies of *PcpsiK* on a Ty-integrative vector (Maury et al., 2016). Previous studies have demonstrated this enzyme can convert psilocin back to psilocybin (Fricke et al., 2017), therefore we hypothesized that increased expression would help shift the equilibrium back towards psilocybin. However, we observed no statistically significant difference in psilocybin or psilocin titers in this strain (ST9334) compared to the parental control (ST9328) (Supplementary Table 4).

### 3.4 Fed-batch fermentation leads to improved psilocybin production

**Fig. 5:**
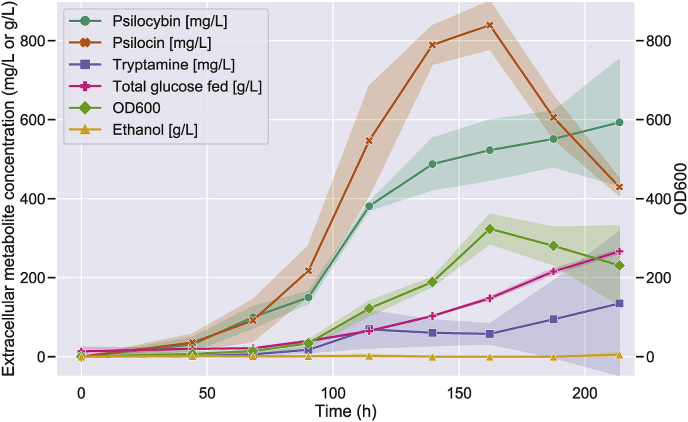
**Controlled fed-batch fermentation results in higher titers.** Production data from fed-batch fermentations of ST9482. Data is presented as averages from triplicate fermentations with standard deviations presented in shaded colours.

To demonstrate industrial applicability of psilocybin production in *S. cerevisiae*, controlled fed-batch fermentation was performed with the top producing strain (ST9482). By cultivating the strain in bioreactors, the pH, oxygen supply and aeration could be accurately controlled and higher glucose amounts could be fed, which should allow for comparatively higher production compared to micro-titer plate cultivation. Fed-batch fermentation was performed in triplicate with an initial exponential feed-rate of 0.02 h^−1^ with a 513 g/L glucose feed media (determined by HPLC analysis of the feed-media). In total, 266.6 ± 5.2 g/L of glucose was fed over a period of 213.7 h. Controlled fed-batch fermentation of ST9482 led to the production of 627 ± 140 mg/L of psilocybin and 580 ± 276 mg/L of psilocin (Fig. 5, Supplementary Fig. 2), increasing the final titer compared to micro-titer plate cultivation in synthetic media. Furthermore, in contrast to batch cultivation in micro-titer plates, due to the controlled feeding of glucose at a low rate, no significant ethanol accumulation was observed.

### 3.5 Production of tryptamine derivatives

**Fig. 6:**
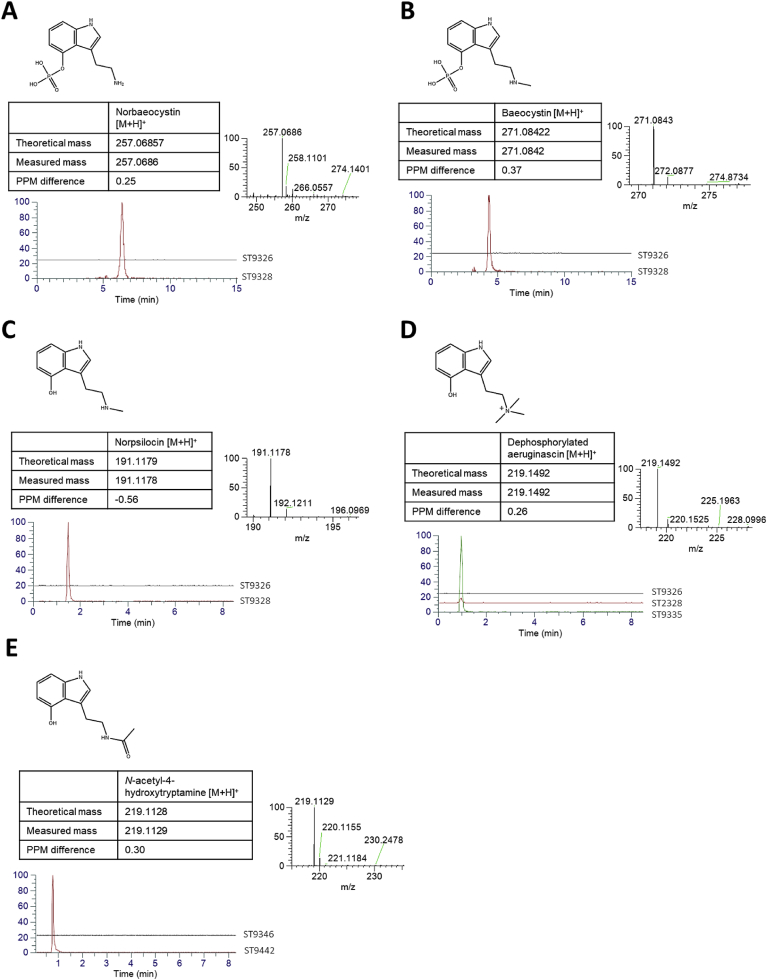
**Production of 4-hydroxytryptamine derivatives and accumulation of psilocybin pathway intermediates in engineered *S. cerevisiae* strains.** LC-MS chromatograms and corresponding mass spectra for (A) Norbaeocystin, (B) Baeocystin, (C) Norpsilocin, (D) Dephosphorylated aeruginascin, and (E) *N*-acetyl-4-hydroxytryptamine produced in engineered *S. cerevisiae* strains ST9326 (Wild-type control), ST9346 (4-hydroxytryptamine control) ST9328 (*Crtdc*, *PcpsiH*, *Pccpr*, *PcpsiK*, *PcpsiM*), ST9335 (*Crtdc*, *PcpsiH*, *Pccpr*, *PcpsiK*, *PcpsiM* multi-copy), ST9442 (*Crtdc*, *PcpsiH*, *Pccpr*, *BtAANAT* multi-copy).

Finally, in an attempt to expand the utility of these engineered strains, we investigated whether *S. cerevisiae* could be engineered to produce natural and new-to-nature tryptamine derivatives. While most enzymes typically display strict substrate specificity, others display relaxed substrate specificity and can accept multiple substrates with various affinities. Given that enzymes of the psilocybin biosynthesis pathway have previously been shown to accept multiple substrates *in vitro*, with *PcPsiK* accepting 4-hydroxytryptamine and psilocin as substrate, and *PcPsiD* (tryptophan decarboxylase from *P. cubensis*) accepting both tryptophan and 4-hydroxytryptophan (Fricke et al., 2017), we postulated that these enzymes could be “coaxed” into accepting other substrates, leading to the production of other potentially interesting products. We first investigated whether only psilocin could be produced by omitting the 4-hydroxytryptamine kinase reaction (*PcPsiK*) (ST9441). Interestingly, omitting this gene abolished all production indicating that *PcPsiM* only accepts phosphorylated 4-hydroxytryptamine substrates (Supplementary Fig. 1). LC-MS analysis of the psilocybin producing strain ST9328 (containing the psilocybin biosynthetic pathway and optimized *Pccpr* expression) tentatively (in the absence of authentic analytical standards) detected the presence of the pathway intermediates norbaeocystin (non N-methylated) (Fig. 6A), baeocystin (mono-N-methylated) (Fig. 6B) and psilocybin (di-N-methylated), as well as their dephosphorylated degradation products psilocin, and norpsilocin (dephosphorylated baeocystin) (Fig. 6C) catalyzed by *PcPsiM*. We then postulated that this enzyme could catalyze a third iterative N-methylation to produce the tri-N-methylated derivative aeruginascin (Jensen et al., 2006). Interestingly, while we could not observe a peak matching the expected m/z of aeruginascin, a peak matching the expected m/z of the dephosphorylated version of aeruginascin was detected in ST9328. Furthermore, introduction of multiple copies of *PcpsiM* using a Ty-integrative vector (ST9335) led to a 21-fold increase in the dephosphorylated aeruginascin peak area (Fig. 6D, Supplementary Table 4), thereby strongly corroborating the production of the tri-*N*-methylated derivative. Finally, to demonstrate the production of new-to-nature derivatives, we investigated whether serotonin-N-acetyl transferase from *Bos taurus* (*BtAANAT*), previously demonstrated to convert 5-hydroxytryptamine (serotonin) into N-acetyl serotonin (normelatonin) in *S. cerevisiae* (Germann et al., 2016) could also accept 4-hydroxytryptamine. Introducing a Ty-integrative vector into a strain that only produces 4-hydroxytryptamine (ST9346) led to the detection of a peak matching the expected m/z of *N*-acetyl-4-hydroxytryptamine in the constructed strain (ST9442) demonstrating not only the relaxed substrate specificity of *BtAANAT*, but also the successful production of a novel molecule structurally similar to both psilocin and normelatonin with potentially novel pharmacological activity (Fig. 6E).

## 4 Discussion

In this study, high-level production of psilocybin was achieved in the industrial workhorse *Saccharomyces cerevisiae*, demonstrating a feasible option for the future production of this pharmaceutically interesting molecule. While introducing the basic biosynthetic pathway into yeast led to the production of psilocybin, high-level production, and corresponding consumption of excess tryptamine was achieved only upon expression of a putative cytochrome P450 reductase (*Pccpr*) from the host species *P. cubensis*. Functional expression was also dependent on the level of *Pccpr* transcription, with expression from the strong constitutive promoter pTEF1 resulting in an increase in psilocybin titers (ST9328) while expression from the mid-strength constitutive promoter pTEF2 resulted in only a small increase in psilocybin titers (ST9329) (Fig. 4A). Overall, this indicates that not only is a species-specific CPR required for functional expression of this tryptamine 4-monooxygenase, but the ratio of CYP to CPR is also important. The finding that the *S. cerevisiae* CPR (NCP1) expressed both from its native promoter and strong constitutive promoter pTEF1 as well as *A. thaliana* CPR (*AtAtr2*) are both poorly compatible with the *P. cubensis* CYP (*PcPsiH*) is completely in-line with previous studies and demonstrates the importance of investigating the CYP:CPR interaction carefully (Renault et al., 2014). Interestingly, functional expression of *PcpsiH* required strong constitutive expression of *Pccpr* from pTEF1. Excess expression of CPR's are often toxic to the cell as it leads to electron transfer uncoupling and the production of reactive oxygen species (ROS). For this reason, most native and heterologous CYP enzymes are expressed far in excess of their CPR partner (Renault et al., 2014). The observation that strong expression of both CYP and CPR was required for optimal expression in this case could be explained by the fact that, due to the membrane bound localization of these enzymes; many are poorly expressed in a heterologous host (Renault et al., 2014). This further highlights the need to thoroughly explore CYP and CPR expression levels to ensure functional heterologous expression.

Further rational engineering resulted in a successive increase in psilocybin titers. In particular, deletion of *RIC1* produced an 18% increase in titer (ST9179) (Fig. 4B). This gene has been previously shown to regulate the shikimate pathway, and its deletion led to a similar increase in the 3-dehydroshikimate derived product, *cis,cis*-muconic acid (Suástegui et al., 2017) suggesting that this strategy is generally applicable to all products derived from the shikimate pathway. While further overexpression of native shikimate pathway genes *ARO1* and *ARO2* led to a modest increase in production (ST9316), a further substantial increase was achieved by introducing multiple copies of *PcpsiM* (ST9492) (Fig. 4B) indicating a clear rate-limiting step and overall suggests that future engineering efforts should focus on improving the catalytic activity of this enzyme. The decrease in titer observed after overexpression of feedback insensitive *Aro4*^*K229L*^*, Trp2*^*S65R, S76L*^ (ST9318) may be explained by the increased accumulation of pathway intermediates baeocystin and norbaeocystin and their possible negative impact on cell physiology. Indeed a significant decrease in final OD600 was observed for this strain which subsequently increased after introducing multiple copies of *PcpsiM* (data not shown) and observing a decrease in the LC-MS peak areas of baeocystin and norbaeocystin (Supplementary Table 4). While integration of multiple copies of *PcpsiM* by a Ty-integrative vector may have resulted in an improved production, for large-scale fermentation this will not be a viable solution to overcoming rate-limiting steps due to previously observed genome instability of Ty-integrations (Maury et al., 2016). However, since Ty-integrative vectors can introduce a large number of genes into the genome in a single transformation step, it serves as a rapid method for demonstrating where sub-optimal reaction steps are in a pathway of interest. Further development of this strain will require either stable, targeted integration of more copies of *PcpsiM,* or enzyme engineering to improve the catalytic efficiency of this reaction.

Fed-batch fermentation of the top producing strain (ST9482) provided a proof-of-concept for industrial-scale production, and while separation and purification of psilocybin from the fermentation broth were not investigated in this work, the observation that psilocybin was efficiently excreted into the extracellular space by yeast will facilitate a simple and cost-effective downstream processing procedure. While we were able to demonstrate high-level psilocybin production (627 ± 140 mg/L in fed-batch fermentation), this was accompanied by concomitant production of psilocin (580 ± 276 mg/L). In fact, significant accumulation of psilocin was observed in all strains tested (Fig. 4. Fig. 5. Supplementary Table 4, Supplementary Fig. 2) indicating a strong equilibrium reaction between the two species. *S. cerevisiae* contains multiple phosphatase enzymes capable of removing phosphate groups from proteins and small-molecules (Offley and Schmidt, 2019; Oshima, 1997), which may be responsible for the conversion of psilocybin to psilocin. Psilocybin is also known to spontaneously degrade to psilocin under acidic conditions (Horita and Weber, 1962). Indeed we observed a small amount of psilocin from an authentic psilocybin standard kept at neutral pH and room temperature for 1h (data not shown) indicating that even under these mild conditions, spontaneous degradation can occur. This is further supported by the detection of dephosphorylated versions of baeocystin (norpsilocin) and aeruginascin. While this psilocybin-psilocin equilibrium reaction is driven on one side by enzymatic or spontaneous degradation, it is assumed that conversion of psilocin back to psilocybin is mediated by *PcPsiK*. Indeed (Fricke et al., 2017) showed that *PcPsiK* could phosphorylate both 4-hydroxytryptamine and psilocin *in vitro.* During fed-batch fermentation of the top psilocybin producing strain ST9482 a decrease in psilocin and corresponding increase in psilocybin titer was observed after 139h (Fig. 5) suggesting that *in vivo PcPsiK* is catalyzing the conversion of psilocin back to psilocybin. However, the detection of dephosphorylated versions of baeocystin (norpsilocin) and aeruginascin (Fig. 6), and the observation that introduction of several copies of *PcpsiK* made no statistically significant change in psilocybin or psilocin titers (Supplementary Table 4), suggests that (de)phosphorylation of these molecules may be more complex than initially thought. Given that current clinical trials are focusing on psilocybin and not the dephosphorylated product, overcoming this problem and preventing psilocin accumulation is a key challenge that must be addressed before commercial production can be realized. At this early stage it can only be speculated why phosphorylated psilocybin is more pharmacologically relevant than non-phosphorylated psilocin, however given that psilocybin is more soluble than psilocin, and that psilocin is predominantly excreted from the body via *O-*glucuronidation (Dinis-Oliveira, 2017), it can be proposed that phosphorylation is an important mechanism for improving the pharmacokinetics of this molecule.

While psilocybin is an interesting and pharmaceutically relevant molecule in its own right, one of the unique features of synthetic biology is the ability to “mix and match” enzymes to create new interesting molecules that aren't found in nature and are infeasible to produce by chemical synthesis. Tryptamine derivatives are an interesting class of molecule that are known to exert their effect through interaction with different serotonin receptors in the body (Glennon and Gessner, 1979; Nichols, 2012). While many are known primarily for their psychoactive effect, other molecules in this class are recognized and in some cases used as anti-depressants (e.g. α-methyltryptamine and α-ethyltryptamine), antimicrobials (e.g. 6-bromo-8,1′-dihydro-isoplysin A), anthelmintics (e.g. convolutindole A) sleep aids (e.g. melatonin), sedatives (e.g. 5-Bromo-DMT) and anti-migraine drugs (e.g. Sumatriptan and other triptan drugs) (Campos et al., 2019; Greene, 2013; Kousara et al., 2017; Tittarelli et al., 2014). In this study, we explored the possibility of using *S. cerevisiae* to produce derivatives from 4-hydroxylated substrates. It was found that omitting *PcPsiK* resulted in no production of psilocybin or psilocin (Supplementary Fig. 1) indicating that the phosphate group is essential for recognition and catalytic conversion by *PcPsiM* as previously reported *in vitro* (Fricke et al., 2019). Our efforts resulted in the production of several 4-hydroxytryptamine derivatives, including intermediates in the psilocybin biosynthetic pathway, and in particular, (dephosphorylated) aeruginascin (ST9335) and N-acetyl-4-hydroxytryptamine (ST9442). Aeruginascin is produced by a third iterative *N*-methylation by an *N*-methyltransferase and is found predominantly in the psychedelic mushroom species *Inocybe aeruginascens* (Jensen et al., 2006). Interestingly, in this work dephosphorylated aeruginascin was produced by introducing multiple copies of *PcpsiM*, even though it has never been detected in *Psilocybe cubensis*, suggesting its production may simply be a matter of increasing the activity of the *N*-methyltransferase enzyme. Little research has been performed with this molecule, primarily due to difficulties in obtaining a pure source either by chemical synthesis or extraction from a natural source. It is however described as producing a euphoric instead of hallucinogenic effect upon ingestion (Gartz, 1989). This difference in effect is hypothesized to be due to the inability of aeruginascin to cross the blood-brain barrier (Jensen et al., 2006), and thereby offers an interesting avenue for drug discovery as a way to separate potential therapeutic effects from potentially unwanted psychotropic effects. While we could only detected the dephosphorylated version of aeruginascin in engineered *S. cerevisiae* strains, given the strict substrate specificity observed for *PcPsiM* for phosphorylated substrates, it can be assumed that the phosphorylated version was produced but was subsequently removed by phosphatases or by spontaneous degradation. The complete dephosphorylation of aeruginascin may be influenced by the positive charge of its quaternary amide group and/or by the substrate specificity of *PsPsiK* and its ability to (re-)phosphorylate alternate substrates. Production of *Psilocybe* derived tryptamine molecules in *S. cerevisiae* further offers the possibility of isolating useful quantities of these molecules in high purity thereby facilitating increased investigation into their *in vivo* function, which, so far has been severely limited by their availability.

*N*-acetyl-4-hydroxytryptamine was produced by introducing a serotonin *N*-acetyltransferase into a 4-hydroxytryptamine producing strain (ST9442) thereby resulting in a (to our knowledge) novel molecule structurally similar to the human neurotransmitter *N*-acetylserotonin (normelatonin) with the hydroxyl group on the 4 position instead of the 5 position. While it would be premature to speculate on possible pharmacological functions of this new molecule, it demonstrates how *in vivo* enzymatic biosynthesis can be used to create novel structural variants of molecules that would otherwise be too complex to produce by chemical synthesis. Indeed, stereoselective introduction of hydroxyl groups into aromatic rings has been described as “one of the most challenging fields in modern (chemical) synthesis” (Ullrich and Hofrichter, 2007), thereby highlighting the unique potential of engineered *S. cerevisiae* strains in the drug discovery process.

## 5 Conclusions

In this work, the successful production of psilocybin was demonstrated in *Saccharomyces cerevisiae*. Implementation of the biosynthetic pathway from *Psilocybe cubensis* with expression of a novel cytochrome P450 reductase resulted in high-level *de novo* production from glucose. Further rational engineering of native *S. cerevisiae* metabolism demonstrated how improved production can be achieved by boosting the native supply of the pathway precursor tryptophan. Finally, the production of natural and new-to-nature 4-hydroxytryptamine derivatives demonstrates the versatility of enzymatic biosynthesis, and how *S. cerevisiae* can be used to produce novel drug candidates that go beyond what is feasible with chemical synthesis or extraction from natural sources.

## CRediT authorship contribution statement

**N. Milne:** Conceptualization, Methodology, Validation, Investigation, Writing - original draft, Writing - review & editing. **P. Thomsen:** Validation, Investigation, Visualization. **N. Mølgaard Knudsen:** Investigation, Visualization, Validation. **P. Rubaszka:** Validation, Investigation. **M. Kristensen:** Methodology, Validation, Investigation. **I. Borodina:** Funding acquisition, Project administration, Supervision, Writing - original draft.

## Declaration of competing interest

NM and IB are co-inventors on a patent application related to this research.
