# Supplementary material for: Metabolic engineering of Saccharomyces cerevisiae for the de novo production of psilocybin and related tryptamine derivatives
Source: Metab Eng. 2020 Jul;60:25–36. doi: 10.1016/j.ymben.2019.12.007 (PMC7232020; doi:10.1016/j.ymben.2019.12.007)
Supplement: Multimedia component 1 [file mmc1.docx]

**Supplementary Table 1.** Plasmids used in this study

| Name | Relevent characteristics | Origin |
| --- | --- | --- |
|  |  |  |
| Templates for PCR amplification | |  |
|  |  |  |
| p0029 | *pTEF1-pPGK1* fused promoters | (Siavash Partow, Verena Siewers, Sara Bjørn, Jens Nielsen, 2010) |
| p0998 | *pPGK1->BtAANAT* | (Germann et al., 2016) |
| p1977 | *pTDH3-pTEF1* fused promoters | (Jessop-Fabre et al., 2016) |
| p02733 | *ARO1<-pTEF1-pPGK1->ARO2* | This study |
| pCfB8532 | *CYB5<-pPGK1-pTEF1->AtAtr2* | This study |
| pCfB8793 | *CrTdc<-pTEF1-pPGK1->PcPsiH* | This study |
| pCfB8795 | *PcCpr<-pTEF1* | This study |
| pCfB8799 | *PcCpr<-pTEF2* | This study |
| pCfB9075 | *TRP1<-pTDH3-pTEF->TRP3* | This study |
| pCfB9326 | *pTEF1->NCP1* |  |
|  |  |  |
| gRNA plasmids for targeting genomic integration sites by CRISPR-Cas9 | | |
|  |  |  |
| pCfB3041 | 2μm ori NatMX pSNR52-X-3 gRNA-tSUP4 | (Jessop-Fabre et al., 2016) |
| pCfB3042 | 2μm ori NatMX pSNR52-X-3 gRNA-tSUP4 | (Jessop-Fabre et al., 2016) |
| pCfB3043 | 2μm ori NatMX pSNR52-XI-1- gRNA-tSUP4 | (Jessop-Fabre et al., 2016) |
| pCfB3045 | 2μm ori NatMX pSNR52-XI-3 gRNA-tSUP4 | (Jessop-Fabre et al., 2016) |
| pCfB3048 | 2μm ori NatMX pSNR52-XII-2 gRNA-tSUP4 | (Jessop-Fabre et al., 2016) |
| pCfB3049 | 2μm ori NatMX pSNR52-XII-4 gRNA-tSUP4 | (Jessop-Fabre et al., 2016) |
| pCfB3050 | 2μm ori NatMX pSNR52-XII-5- gRNA-tSUP4 | (Jessop-Fabre et al., 2016) |
| pCfB8783 | 2μm ori NatMX pSNR52-RIC1- gRNA-tSUP4 | This study |
|  |  |  |
| Episomal yeast expression plasmids | |  |
|  |  |  |
| pCfB2312 | 2μm ori *pTEF1->*c*as9* KanMX | (Jessop-Fabre et al., 2016) |
| pTAJAK-71 | 2μm ori NatMX | (Jessop-Fabre et al., 2016) |
|  |  |  |
| Backbone plasmids for EasyClone-MarkerFree plasmid assembly | | |
|  |  |  |
| pCfB255 | pX-2-USER Ura3 | (Jensen et al., 2014) |
| pCfB2903 | pXI-2-USER | (Jessop-Fabre et al., 2016) |
| pCfB2904 | pXI-3-USER | (Jessop-Fabre et al., 2016) |
| pCfB2909 | pXII-5-USER | (Jessop-Fabre et al., 2016) |
| pCfB3040 | pXII-4-USER | (Jessop-Fabre et al., 2016) |
| pCfB3034 | pX-3-USER | (Jessop-Fabre et al., 2016) |
| pCfB8221 | pTy-4-USER pKlURA3(10bp)-KlURA3 | This study |
|  |  |  |
| Plasmids for integration into yeast genome | |  |
|  |  |  |
| pCfB255 | *X-2:: loxP-KlURA3-loxP* | (Jensen et al., 2014) |
| pCfB8794 | *XII-5:: PcPsiM<-pTDH3-pTEF1->PcPsiK* | This study |
| pCfB8796 | *Ty-4:: PcPsiM<-pTDH3-pKlURA3(10bp)->KlURA3* | This study |
| pCfB8881 | *XI-3:: CrTdc<-pTEF1* | This study |
| pCfB9013 | *Ty-4: pPGK1->PcPsiK-pKlURA3(10bp)->KlURA3* | This study |
| pCfB9073 | *XI-1:: PcCpr<-pTEF1-pPGK1->PcPsiH* | This study |
| pCfB9074 | *XII-4:: ARO4^K229L^<-pTDH3-pTEF1->TRP2^S65R,S76L^* | This study |
| pCfB9111 | *Ty-4: pPGK1->BtAANAT-pKlURA3(10bp)->KlURA3* | This study |
| pCfB9153 | *X-2:: loxP-KlURA3-loxP TRP4<-pTDH3-pTEF1-TRP5* | This study |
|  |  |  |

**Supplementary Table 2.** Biobricks used in this study.

| Name | Description | Fwd primer | Rev primer | Template |
| --- | --- | --- | --- | --- |
|  |  |  |  |  |
| BB0008 | TEF1 promoter | PR-5 | PR-6 | p0029 |
| BB0009 | PGK1 promoter | PR-7 | PR-8 | p0029 |
| BB0010 | TEF1-PGK1 fused promoters | PR-5 | PR-8 | p0029 |
| BB0410 | TDH3 promoter | PR-1852 | PR-1853 | p1977 |
| BB0464 | TDH3-TEF1 fused promoters | PR-1853 | PR-1565 | p1977 |
| BB0777 | TEF2 promoter | PR-9694 | PR-9695 | *S. cerevisiae* gDNA |
| BB3816 | *CrTdc* gene | PR-23893 | PR-23894 | Synthetic DNA (GeneArt) |
| BB3817 | *PcPsiH* gene | PR-23895 | PR-23896 | Synthetic DNA (GeneArt) |
| BB3818 | *PcPsiK* gene | PR-23897 | PR-23898 | Synthetic DNA (GeneArt) |
| BB3819 | *PcPsiM* gene | PR-23899 | PR-23900 | Synthetic DNA (GeneArt) |
| BB3820 | *PcCpr* gene | PR-23901 | PR-23902 | Synthetic DNA (GeneArt) |
| BB3923 | *XI-3:: CrTdc<-pTEF1-pPGK1->PcPsiH* integration fragment | PR-23822 | PR-23823 | pCfB8793 |
| BB3939 | *XI-1:: PcCpr<-pTEF1* integration fragment | PR-24259 | PR-24260 | pCfB8795 |
| BB3940 | *XI-1:: PcCpr<-pTEF2* integration fragment | PR-24259 | PR-24260 | pCfB8799 |
| BB3953 | *XI-1:: CYB5<-pPGK1-pTEF1->AtAtr2* integration fragment | PR-24259 | PR-24260 | pCfB8532 |
| BB4090 | *XII-2:: TRP1<-pTDH3-pTEF->TRP3* integration fragment | PR-24893 | PR-24894 | pCfB9075 |
| BB4020 | *X-4:: ARO1<-pTEF1-pPGK1->ARO2* integration fragment | PR-24381 | PR-24382 | p02733 |
| BB4079 | *pPGK1->BtAANAT* overexpression fragment | PR-7 | PR-1762 | p0998 |
| BB4335 | *XI-1:: pTEF1->NCP1* integration fragment | PR-24259 | PR-24260 | pCfB9326 |
|  |  |  |  |  |

**Supplementary Table 3.** Primers used in this study.

| Name | Sequence (5'→3') | Purpose |
| --- | --- | --- |
|  |  |  |
| Primers for biobrick amplification | | |
| PR-5 | ACCTGCACUTTGTAATTAAAACTTAG | Fwd primer to amplify BB0008 and BB0010 |
| PR-6 | CACGCGAUGCACACACCATAGCTTC | Rev primer to amplify BB0008 |
| PR-7 | CGTGCGAUGGAAGTACCTTCAAAGA | Fwd primer to amplify BB0009 |
| PR-8 | ATGACAGAUTTGTTTTATATTTGTTG | Rev primer to amplify BB0009 and BB0010 |
| PR-1565 | ATGACAGAUTTGTAATTAAAACTTAG | Fwd primer to amplify BB0464 |
| PR-1762 | CACGCGAUTTAACGATCGCTATTACGACGCAGTG | Rev primer to amplify BB4079 |
| PR-1852 | CACGCGAUATAAAAAACACGCTTTTTCAG | Fwd primer to amplify BB0410 |
| PR-1853 | ACCTGCACUTTTGTTTGTTTATGTGTGTTTATTC | Rev primer to amplify BB0410 and BB0464 |
| PR-9694 | CACGCGAUATATATGGGGCCGTATAC | Fwd primer to amplify BB0777 |
| PR-9695 | ACCTGCACUGTTTAGTTAATTATAGTTCGTTGAC | Rev primer to amplify BB0777 |
| PR-23822 | GACACATCTAACTGATTAGTTTTCCGTTTTAGGATATTGACGCCAAGCGTGCGTCTGATTGAGCGACCTCATGCTATACCTGAG | Fwd primer to amplify BB3923 and BB4324 |
| PR-23823 | ACTAACATCATGTACAAAACAGTTTAATAATGATCTGTATTGCTGGCTCAATCCACGTAACTTCGAGCGTCCCAAAACCTTCTC | Rev primer to amplify BB3923 and BB4324 |
| PR-23893 | AAAACAATGGGTTCTATTGATTCTACCAACG | Fwd primer to amplify BB3816 |
| PR-23894 | CGTGCGAUTCAGGCTTCTTTCAACAAGTC | Rev primer to amplify BB3816 |
| PR-23895 | ATCTGTCAUAAAACAATGATCGCTGTTTTGTTCTCTTTC | Fwd primer to amplify BB3817 |
| PR-23896 | CACGCGAUTTATGGACCAGAAACAGATTG | Rev primer to amplify BB3817 |
| PR-23897 | ATCTGTCAUAAAACAATGGCTTTCGACTTGAAGACTG | Fwd primer to amplify BB3818 |
| PR-23898 | CACGCGAUTTAAGCAGTAGAAGATTCCTTCAAC | Rev primer to amplify BB3818 |
| PR-23899 | AGTGCAGGUAAAACAATGCACATCAGAAACCCATAC | Fwd primer to amplify BB3819 |
| PR-23900 | CGTGCGAUTTAGAACAAAGAAGACAATTCTGGG | Rev primer to amplify BB3819 |
| PR-23901 | AGTGCAGGUAAAACAATGGCTTCTTCTTCTTCTGACG | Fwd primer to amplify BB3820 |
| PR-23902 | CGTGCGAUTTAAGACCAAACGTCCAACATC | Rev primer to amplify BB3820 |
| PR-24259 | AAACCCGTGCTGAAATCCGTGCACCGCATCAAATTTTCTCGGAGGATTCTTTGCAGCCGGGAGCGACCTCATGCTATACCTGAG | Fwd primer to amplify BB3953, BB3939, BB3940 and BB4335 |
| PR-24260 | GCGGTGTGAGTTTCCGTCTGTACGCAGCATTTAGCAGAGATTTGCCAATGCCAAGAAACTCTTCGAGCGTCCCAAAACCTTCTC | Rev primer to amplify BB3953, BB3939 BB3940 and BB4335 |
| PR-24893 | GCCATTTTTTTTTCTGTATCGGGCCCTCCTTACTGCTCTCCTTCCGTGTAACGCGTTATGGAGCGACCTCATGCTATACCTGAG | Fwd primer to amplify BB4090 |
| PR-24894 | AAGTGGCAAAAGCGTTAGACGCAGTACAAGGACGCGTTAAGAAAAATTTCGAGAGAGTCGCTTCGAGCGTCCCAAAACCTTCTC | Rev primer to amplify BB4090 |
| PR-24381 | CCAACTACCAAGGTTGTTGAGGGAACACTGGGGCAATAGGCTGTCGCCATTCAAGAGCAGGAGCGACCTCATGCTATACCTGAG | Fwd primer to amplify BB4020 |
| PR-24382 | TTCTTGCAGACATCAGACATACTATTGTAATTCAAAAAAAAAAAGCGAATCTTCCCATGTCTTCGAGCGTCCCAAAACCTTCTC | Rev primer to amplify BB4020 |
|  |  |  |
| Primers for verification of correct EasyClone plasmid assembly | | |
|  |  |  |
| PR-22955 | GACGGTAGGTATTGATTGTAATTCTG | pTDH3 Fwd diagnostic PCR primer |
| PR-339 | GCTCATTAGAAAGAAAGCATAGC | pTEF1 Fwd diagnostic PCR primer |
| PR-340 | TACAGATCATCAAGGAAGTAATTATC | pPGK1 Fwd diagnostic PCR primer |
| PR-224 | GAAATTCGCTTATTTAGAAGTGTC | tADH1 Rev diagnostic PCR primer |
| PR-225 | CTCCTTCCTTTTCGGTTAGAG | tCYC1 Rev diagnostic PCR primer |
| PR-23875 | ACTGTTGGGAAGGGCGATC | gRNA cassette Fwd diagnostic PCR primer |
| PR-23876 | AGCGCCCAATACGCAAAC | gRNA cassette Rev diagnostic PCR primer |
|  |  |  |
| Primers for genotyping correct genomic integration of expression cassettes | | |
|  |  |  |
| PR-2221 | GTTGACACTTCTAAATAAGCGAATTTC | Universal Rev primer binding in *S. cerevisiae* integration cassettes |
| PR-893 | CGAAGAAGGCCTGCAATTC | Fwd primer for diagnostic PCR of EasyClone plasmid integration at XII-2 site |
| PR-894 | GGCCCTGATAAGGTTGTTG | Rev primer for diagnostic PCR of EasyClone plasmid integration at XII-2 site |
| PR-897 | GAACTGACGTCGAAGGCTCT | Fwd primer for diagnostic PCR of EasyClone plasmid integration at XII-4 site |
| PR-898 | CGTGAAATCTCTTTGCGGTAG | Rev primer for diagnostic PCR of EasyClone plasmid integration at XII-4 site |
| PR-899 | CCACCGAAGTTGATTTGCTT | Fwd primer for diagnostic PCR of EasyClone plasmid integration at XII-5 site |
| PR-900 | GTGGGAGTAAGGGATCCTGT | Rev primer for diagnostic PCR of EasyClone plasmid integration at XII-5 site |
| PR-905 | CTCACAAAGGGACGAATCCT | Fwd primer for diagnostic PCR of EasyClone plasmid integration at X-4 site |
| PR-906 | GACGGTACGTTGACCAGAG | Rev primer for diagnostic PCR of EasyClone plasmid integration at X-4 site |
| PR-907 | CTTAATGGGTAGTGCTTGACACG | Fwd primer for diagnostic PCR of EasyClone plasmid integration at XI-1 site |
| PR-908 | GAAGACCCATGGTTCCAAGGA | Rev primer for diagnostic PCR of EasyClone plasmid integration at XI-1 site |
| PR-911 | GTGCTTGATTTGCGTCATTC | Fwd primer for diagnostic PCR of EasyClone plasmid integration at XI-3 site |
| PR-912 | CACATTGAGCGAATGAAACG | Rev primer for diagnostic PCR of EasyClone plasmid integration at XI-3 site |
| PR-23853 | CAGCACTTATATCCGCATCAAACTC | Fwd primer for diagnostic PCR of RIC1 gene knockout |
| PR-23854 | CTTGATCAGCCATGCCTATTATACTG | Rev primer for diagnostic PCR of RIC1 gene knockout |
|  |  |  |
| gRNA repair dsDNA oligos | | |
|  |  |  |
| PR-23852 | AGGCTTTTGTTCTTTGATGTTAATTCGGCAATACCGACGCCTTTGATCTATATGACACCATGTATTCTTTACCATATAGCTTCATAACAT | dsDNA oligo repair fragment for RIC gene deletion |
|  |  |  |

**Supplementary Table 4.** Collected LC-MS data from psilocybin and tryptamine derivative producing strains analyzed in this study. Data is presented either as peak area or as concentration (in mg/L) when peak areas could be quantified using authentic analytical standards. Data is presented as averages and standard deviations from two biological replicates. BDL; Below detection limit, ND; Not determined.

| Strain name | Psilocybin | Psilocin | Tryptamine | Dephos. Aeruginascin | Norpsilocin | Baeocystin | Norbaeocystin | *N*-acetyl-4-hydroxytryptamine |
| --- | --- | --- | --- | --- | --- | --- | --- | --- |
|  | (mg/L) | (mg/L) | (mg/L) | (Peak area) | (Peak area) | (Peak area) | (Peak area) | (Peak area) |
| ST9326 | BDL | BDL | BDL | BDL | BDL | BDL | BDL | BDL |
| ST9327 | 2.2 ± 0.7 | 5.5 ± 0.7 | 120.3 ± 11.1 | 2688168.2 ± 947522 | 22312402.1 ± 5567549.2 | BDL | BDL | BDL |
| ST9328 | 137.1 ± 8.3 | 82.8 ± 3.7 | 13.3 ± 1.0 | 2798416.1 ± 492879.4 | 144714921.0 ± 4874438.4 | 5170134.1 ± 143406.0 | 6316663,4 ± 432794.1 | BDL |
| ST9329 | 12.6 ± 0.8 | 21.0 ± 2.3 | 0.8 ± 0.1 | ND | ND | 34856.8 ± 4304.6 | BDL | ND |
| ST9330 | 0.04 ± 0.00 | 0.03 ± 0.00 | 165,3 ± 8.6 | ND | ND | BDL | BDL | ND |
| ST9179 | 162.1 ± 10.6 | 87.9 ± 5.1 | 2.8 ± 0.3 | ND | ND | 2839987.5 ± 365762.6 | 3244250.4 ± 424160.6 | ND |
| ST9316 | 178.6 ± 2.7 | 79.8 ± 2.7 | 4.1 ± 1.2 | ND | ND | 3379592.5 ± 24429.5 | 3810274.9 ± 87445.4 | ND |
| ST9318 | 141.1 ± 12.5 | 33.2 ± 2.0 | 54.9 ± 0.1 | ND | ND | 19115929.9 ± 11494.3 | 12772941.9 ± 593810.8 | ND |
| ST9441 | BDL | BDL | 34.8 ± 0.2 | BDL | BDL | BDL | BDL | ND |
| ST9335 | 121.9 ± 14.8 | 81.8 ± 5.3 | 1.7 ± 0.3 | 28109046.6 ± 1348166.2 | 9281833.7 ± 1373680.4 | 1838226.5 ± 94265.2 | 1018688.5 ± 31893.0 | ND |
| ST9442 | ND | ND | ND | ND | ND | ND | ND | 122459804.3 ± 24769687.1 |
| ST9482 | 200.5 ± 6.5 | 31.4 ± 0.1 | 68.9 ± 1.3 | ND | ND | 1787344.0 ± 19912.5 | 1546709.4 ± 130917.1 | ND |
| ST9334 | 95.7 ± 1.5 | 108.2 ± 2.0 | 1.5 ± 0.1 | ND | ND | ND | ND | ND |
| ST9649 | 8.3 ± 0.3 | 11.1 ± 0.6 | 104.5 ± 5.9 | ND | ND | ND | ND | ND |


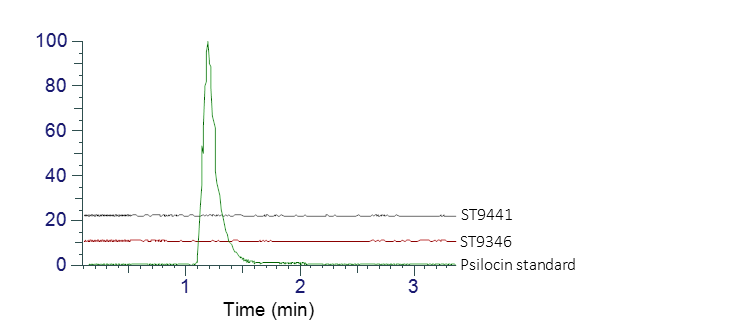


**Supplementary Figure 1.** ***PcPsiM* is specific for phosphorylated 4-hydroxytryptamine substrates.** LC-MS chromatogram showing the absence of psilocin production in strains lacking *PcPsiK*. ST9346; 4-hydroxytryptamine producing parental strain (*Crtdc*, *PcpsiH*, *Pccpr*). ST9346; Strain additionally expressing *PcpsiM* (*Crtdc*, *PcpsiH*, *Pccpr*, *PcpsiM* multi-copy).

**Supplementary Figure 2.** DNA coding sequence of *Psilocybe cubensis* cytochrome P450 reductase (*Pccpr*) codon optimized for expression in *S. cerevisiae*.

ATGGCTTCTTCTTCTTCTGACGTTTTCGTTTTGGGTTTGGGTGTTGTTTTGGCTGCTTTGTACATCTTCAGAGACCAATTGTTCGCTGCTTCTAAGCCAAAGGTTGCTCCAGTTTCTACTACTAAGCCAGCTAACGGTTCTGCTAACCCAAGAGACTTCATCGCTAAGATGAAGCAAGGTAAGAAGAGAATCGTTATCTTCTACGGTTCTCAAACTGGTACTGCTGAAGAATACGCTATCAGATTGGCTAAGGAAGCTAAGCAAAAGTTCGGTTTGGCTTCTTTGGTTTGTGACCCAGAAGAATACGACTTCGAAAAGTTGGACCAATTGCCAGAAGACTCTATCGCTTTCTTCGTTGTTGCTACTTACGGTGAAGGTGAACCAACTGACAACGCTGTTCAATTGTTGCAAAACTTGCAAGACGAATCTTTCGAATTCTCTTCTGGTGAAAGAAAGTTGTCTGGTTTGAAGTACGTTGTTTTCGGTTTGGGTAACAAGACTTACGAACACTACAACTTGATCGGTAGAACTGTTGACGCTCAATTGGCTAAGATGGGTGCTATCAGAATCGGTGAAAGAGGTGAAGGTGACGACGACAAGTCTATGGAAGAAGACTACTTGGAATGGAAGGACGGTATGTGGGAAGCTTTCGCTACTGCTATGGGTGTTGAAGAAGGTCAAGGTGGTGACTCTGCTGACTTCGTTGTTTCTGAATTGGAATCTCACCCACCAGAAAAGGTTTACCAAGGTGAATTCTCTGCTAGAGCTTTGACTAAGACTAAGGGTATCCACGACGCTAAGAACCCATTCGCTGCTCCAATCGCTGTTGCTAGAGAATTGTTCCAATCTGTTGTTGACAGAAACTGTGTTCACGTTGAATTCAACATCGAAGGTTCTGGTATCACTTACCAACACGGTGACCACGTTGGTTTGTGGCCATTGAACCCAGACGTTGAAGTTGAAAGATTGTTGTGTGTTTTGGGTTTGGCTGAAAAGAGAGACGCTGTTATCTCTATCGAATCTTTGGACCCAGCTTTGGCTAAGGTTCCATTCCCAGTTCCAACTACTTACGGTGCTGTTTTGAGACACTACATCGACATCTCTGCTGTTGCTGGTAGACAAATCTTGGGTACTTTGTCTAAGTTCGCTCCAACTCCAGAAGCTGAAGCTTTCTTGAGAAACTTGAACACTAACAAGGAAGAATACCACAACGTTGTTGCTAACGGTTGTTTGAAGTTGGGTGAAATCTTGCAAATCGCTACTGGTAACGACATCACTGTTCCACCAACTACTGCTAACACTACTAAGTGGCCAATCCCATTCGACATCATCGTTTCTGCTATCCCAAGATTGCAACCAAGATACTACTCTATCTCTTCTTCTCCAAAGATCCACCCAAACACTATCCACGCTACTGTTGTTGTTTTGAAGTACGAAAACGTTCCAACTGAACCAATCCCAAGAAAGTGGGTTTACGGTGTTGGTTCTAACTTCTTGTTGAACTTGAAGTACGCTGTTAACAAGGAACCAGTTCCATACATCACTCAAAACGGTGAACAAAGAGTTGGTGTTCCAGAATACTTGATCGCTGGTCCAAGAGGTTCTTACAAGACTGAATCTTTCTACAAGGCTCCAATCCACGTTAGAAGATCTACTTTCAGATTGCCAACTAACCCAAAGTCTCCAGTTATCATGATCGGTCCAGGTACTGGTGTTGCTCCATTCAGAGGTTTCGTTCAAGAAAGAGTTGCTTTGGCTAGAAGATCTATCGAAAAGAACGGTCCAGACTCTTTGGCTGACTGGGGTAGAATCTCTTTGTTCTACGGTTGTAGAAGATCTGACGAAGACTTCTTGTACAAGGACGAATGGCCACAATACGAAGCTGAATTGAAGGGTAAGTTCAAGTTGCACTGTGCTTTCTCTAGACAAAACTACAAGCCAGACGGTTCTAAGATCTACGTTCAAGACTTGATCTGGGAAGACAGAGAACACATCGCTGACGCTATCTTGAACGGTAAGGGTTACGTTTACATCTGTGGTGAAGCTAAGTCTATGTCTAAGCAAGTTGAAGAAGTTTTGGCTAAGATCTTGGGTGAAGCTAAGGGTGGTTCTGGTCCAGTTGAAGGTGTTGCTGAAGTTAAGTTGTTGAAGGAAAGATCTAGATTGATGTTGGACGTTTGGTCTTAA

Germann, S.M., Baallal Jacobsen, S.A., Schneider, K., Harrison, S.J., Jensen, N.B., Chen, X., Stahlhut, S.G., Borodina, I., Luo, H., Zhu, J., Maury, J., Forster, J., 2016. Glucose-based microbial production of the hormone melatonin in yeast Saccharomyces cerevisiae. Biotechnol. J. 11, 717–724. https://doi.org/10.1002/biot.201500143

Jensen, N.B., Strucko, T., Kildegaard, K.R., David, F., Maury, J., Mortensen, U.H., Forster, J., Nielsen, J., Borodina, I., 2014. EasyClone: Method for iterative chromosomal integration of multiple genes in Saccharomyces cerevisiae. FEMS Yeast Res. 14, 238–248. https://doi.org/10.1111/1567-1364.12118

Jessop-Fabre, M.M., Jakočiūnas, T., Stovicek, V., Dai, Z., Jensen, M.K., Keasling, J.D., Borodina, I., 2016. EasyClone-MarkerFree: A vector toolkit for marker-less integration of genes into Saccharomyces cerevisiae via CRISPR-Cas9. Biotechnol. J. 11, 1110–1117. https://doi.org/10.1002/biot.201600147

Siavash Partow, Verena Siewers, Sara Bjørn, Jens Nielsen, J.M., 2010. Characterization of different promoters for designing a new expression vector in Saccharomyces cerevisiae. Yeast 27, 955–964. https://doi.org/10.1002/yea
